# Renal Cell Carcinoma in the Geriatric Population: Toward a Frailty-Adapted Therapeutic Paradigm

**DOI:** 10.3390/jcm15176726

**Published:** 2026-08-30

**Authors:** Cemile Ozsurekci, Ufuk Yazar, Yusuf Acikgoz

**Affiliations:** 1 Division of Geriatrics, Department of Internal Medicine, Lokman Hekim University Ankara Hospital, 06930 Ankara, Turkey; 2 Department of Internal Medicine, Lokman Hekim University Ankara Hospital, 06930 Ankara, Turkey; 3 Division of Medical Oncology, Department of Internal Medicine, Lokman Hekim University Ankara Hospital, 06930 Ankara, Turkey

**Keywords:** renal cell carcinoma, older adults, geriatric oncology, frailty, comprehensive geriatric assessment, immunotherapy, targeted therapy

## Abstract

**Background:** Renal Cell Carcinoma (RCC) primarily affects older adults, with incidence increasing with age. Older adults remain underrepresented in clinical trials, and treatment decisions are often extrapolated from younger populations. Age-related factors such as comorbidities, frailty, polypharmacy, and immunosenescence complicate management in the geriatric population. **Methods:** A narrative review of the literature was conducted, focusing on localized and metastatic RCC in older adults. The review comprehensively examined the following topics: surgical interventions, active surveillance strategies, systemic therapies (including immune checkpoint inhibitors, vascular endothelial growth factor tyrosine kinase inhibitors, and hypoxia-inducible factor-2α (HIF-2α) inhibitors), toxicity profiles, and real-world outcomes. **Results:** Managing RCC in older adults requires balancing efficacy with tolerability. For localized disease, surveillance and minimally invasive approaches are suitable given mortality risks. In metastatic RCC, immune checkpoint inhibitor combinations and targeted therapies have improved outcomes, though toxicity may increase in frail patients. Given the favorable safety profile of HIF-2α inhibitors, these agents may play a more substantial clinical role and offer improved therapeutic utility in this patient population. Real-world data show disparities between trial populations and practice, highlighting the need for individualized treatment. **Conclusions:** RCC management in geriatric patients requires a shift from age-based to frailty-informed decisions. Integrating geriatric assessment enables better patient selection and treatment tailoring. Future research should focus on geriatric-adapted trials, biomarker strategies, and de-escalation approaches to improve outcomes while reducing toxicity in this population.

## 1. Epidemiology and Rationale for Geriatric-Focused Management in RCC

Renal cell carcinoma (RCC) remains a significant global health burden, representing approximately 4% of all new adult malignancies [1,2]. Asia (34.8%), Europe (33.5%) and North America (18.3%) account for most cases [1]. The epidemiological landscape of RCC has undergone a dramatic shift towards the geriatric population. The median age at diagnosis is 64–66 years, but the rapid increase in incidence is observed in patients aged 75 years and older [2]. This shift in the RCC patient population is driven by two primary factors: global population aging and the widespread use of cross-sectional imaging, which has led to an increase in incidentally detected small renal masses (SRMs) in asymptomatic older adults [3,4].

While age-standardized mortality rates are declining in many countries due to better management, the overall incidence continues to rise globally. This increase in incidence is driven by obesity, smoking, and increased detection of incidental masses [5]. The population of oldest-old (≥80 years) and frail patients continues to grow with global aging, so traditional age-blind treatment algorithms are increasingly viewed as inadequate. In the literature, there is a growing clinical consensus that chronological age is a poor proxy for physiological resilience. Recent research has focused on frailty and new strategies rooted in the unique biological, pharmacological, and psychosocial landscape of the aging patient. Comprehensive Geriatric Assessment (CGA) plays a central role in this approach. By determining actual physiological fitness, the CGA prevents the undertreatment of fit older adults and the overtreatment of frail patients, balancing cancer control with the preservation of independence and quality of life.

## 2. Methods

This narrative review was conducted through a comprehensive literature search of PubMed/MEDLINE, Embase, and Scopus to identify studies evaluating the management of renal cell carcinoma (RCC) in older adults. The search included articles published between January 1997 and May 2026. The final literature search was performed on 17 May 2026. The search was limited to English-language publications. The literature search was performed manually and based on filtering the available evidence according to keywords, publication date, and publication categories. Search terms included combinations of the following keywords and Medical Subject Headings (MeSHs), where applicable: “renal cell carcinoma,” “older adults,” “geriatric,” “frailty,” “comprehensive geriatric assessment,” “localized renal cell carcinoma,” “metastatic renal cell carcinoma,” “active surveillance,” “nephrectomy,” “immunotherapy,” “immune checkpoint inhibitors,” “tyrosine kinase inhibitors,” “targeted therapy,” “quality of life,” and “real-world evidence.” Articles were selected based on their relevance to the management of RCC in older adults, and priority was given to randomized controlled trials, prospective studies, systematic reviews, meta-analyses, large observational studies, and guideline publications. Given the limited availability of prospective studies specifically involving older patients with RCC, selected retrospective studies and real-world evidence were also included when they provided clinically relevant data. The available evidence was critically synthesized to provide a clinically applicable overview of the current management of localized and metastatic RCC in older adults.

## 3. Aging-Related Determinants Relevant to RCC

### 3.1. Immunosenescence, Altered Tumor Biology and Competing Risks

Immunosenescence is defined as the age-related progressive decline in immune function and is characterized by innate and adaptive immune dysfunction, causing poor vaccination outcomes and increased susceptibility to infection, age-related disease, and malignancies. Impairment in the ability of the immune system to recognize and eliminate malignant cells may contribute to the pathogenesis of RCC in older adults. Research has shown that peritumoral tissue in RCC exhibits a senescent and proinflammatory phenotype that may support tumor progression [6]. Moreover, a senescence-related gene set within the tumor tissue might contribute to a poorer prognosis in the older population [7].

In the geriatric population, the natural history of RCC, especially for small renal masses (SRMs < 4 cm), often follows an indolent course characterized by slower growth rates (often <1 mm/year). Although 20–30% of SRMs may exhibit aggressive behavior, the vast majority are discovered incidentally and show minimal progression over time [8,9,10]. Furthermore, older patients may present with different histological subtypes or less aggressive tumor behaviors [11,12]. These patients are often more likely to die from non-renal causes such as cardiovascular events or secondary malignancies than from RCC. For these individuals, care plans must focus on their overall health and life expectancy, rather than just treating the tumor.

### 3.2. Reduced Physiological Reserve and Pharmacokinetics

Aging is characterized by a progressive decline in organ function, most notably a decline in the glomerular filtration rate (GFR) and hepatic metabolism. This reduced physiological reserve makes older patients significantly more susceptible to the toxicities of systemic therapies. Prospective studies have highlighted pharmacokinetic variability with age, including decreases in liver mass and cytochrome P450 (CYP) enzyme content [13,14]. Because many small-molecule targeted agents used for metastatic RCC (mRCC) (e.g., vascular endothelial growth factor (VEGF)/mammalian target of rapamycin (mTOR) inhibitors) are metabolized through CYP3A4, age-related reductions in metabolic capacity may lead to higher toxicity rates [14,15]. Consequently, a significantly higher proportion of patients aged ≥75 years require dose reductions or treatment interruptions [16].

### 3.3. Psychosocial Factors and Polypharmacy

Beyond biological factors, the psychosocial characteristics of the older population present unique challenges to RCC treatment. Polypharmacy, the use of five or more medications, is a common geriatric syndrome and increases the risk of drug–drug interactions when combined with systemic anticancer therapies. Moreover, the prevalence of cognitive impairment, depression, and social isolation can significantly hinder treatment adherence and recovery [17]. Table 1 summarizes the physiological changes in older adults.

## 4. The Evidence Gap: Limitations in Geriatric RCC Research

As RCC becomes more prevalent among older adults, the clinical evidence base remains focused on younger, fitter populations. The primary challenges in the current literature can be categorized into three key areas:

Underrepresentation in Pivotal Clinical Trials: A fundamental limitation is the systematic exclusion of older adults from the phase III randomized controlled trials (RCTs) that define the standards of care. Historically, trials for Tyrosine Kinase Inhibitors (TKIs) and Immune Checkpoint Inhibitors (ICIs) have utilized strict inclusion criteria—such as preserved organ function and a Performance Status (PS) of 0 or 1. These criteria effectively exclude the real-world older patients who have multimorbidity [19,20]. Recent meta-analyses of major ICI-based trials (e.g., CheckMate 9ER, CLEAR, KEYNOTE-426) suggest that while younger patients (<65 years) derive a clear overall survival (OS) benefit from combination therapies, the benefit-risk ratio in patients aged ≥75 years remains less definitive and often fails to reach statistical significance in age-stratified subgroups [21,22,23,24].

Scarcity of Geriatric-Specific Endpoints: The current RCC literature prioritizes oncological endpoints like Progression-Free Survival (PFS) and Objective Response Rate (ORR). However, for older adults, these metrics may be secondary to “functional endpoints,” such as the preservation of Activities of Daily Living (ADLs), cognitive stability, and quality of life. There is a notable lack of prospective data regarding Patient-Reported Outcome Measures (PROMs) or Quality of Life (QoL) specifically validated for frail older adults, which could be helpful for shared decision-making during treatment.

Challenges in Standardizing Real-World Data: While retrospective studies have attempted to fill these gaps using large databases, they are frequently limited by selection bias. Most studies included only the healthiest older adults for aggressive therapy, and there is a lack of data on frail and sarcopenic adults. Frailty may be significantly associated with poorer survival outcomes in patients with RCC and only eight studies examined the association between frailty and survival [17]. Without standardized reporting of frailty in large-scale datasets, the true impact of biological aging on treatment outcomes is masked, with chronological age acting as a misleading confounding variable.

## 5. Geriatric Assessment and Frailty Stratification

### 5.1. Limitations of Standard Performance Status Scales

Standard tools for assessing treatment eligibility, such as the Eastern Cooperative Oncology Group (ECOG) and Karnofsky Performance Status (KPS) scales, have long been the gold standard for determining whether a patient is fit for systemic therapy. However, these metrics lack the sensitivity required to evaluate biological aging in geriatric RCC cohorts. Research indicates that traditional performance status measures often overestimate a frail patient’s resilience, increasing the incidence of iatrogenic toxicity and over-treatment [25,26,27]. In patients with high frailty scores, these scales lose their prognostic value, failing to predict the high rates of toxicity and early treatment discontinuation [27]. The ASCO geriatric oncology guideline recommends formal geriatric assessment because routine measures such as ECOG scores demonstrate insufficient predictive value for treatment-related toxicity [28].

Older patients with an ECOG PS of 0 or 1 may still have subclinical vulnerabilities, including occult sarcopenia or mild cognitive dysfunction, which independently correlate with severe toxicities during immunotherapy or systemic chemotherapy regimens [29,30]. Recent data from a general geriatric oncology population, rather than an RCC-specific cohort, suggest that up to 35% of older cancer patients categorized as “fit” by ECOG PS are identified as “frail” or “vulnerable” when assessed using objective geriatric screening tools, highlighting a substantial risk of over-treatment in this demographic group [31].

### 5.2. Screening Tools (G8, Clinical Frailty Scale)

Given the time constraints of clinical practice, validated screening tools are essential to identify patients who may benefit from a full CGA.

The G8 Screening Tool: This is one of the most commonly used frailty instruments in geriatric oncology practice. It consists of eight items evaluating nutritional status, mobility, neuropsychological problems, and self-rated health. A score of ≤14 indicates the need for a full CGA. In older patients with RCC, the G8 has demonstrated high sensitivity for detecting vulnerable individuals, although its specificity can be limited by the high baseline prevalence of comorbidities in renal cancer [32].

The Clinical Frailty Scale (CFS): This is another widely used geriatric assessment instrument in daily clinical practice. It is a simple 9-point, judgment-based visual scale ranging from 1 (“Very Fit”) to 9 (“Terminally Ill”). The CFS was originally developed to summarize the multidimensional components of a comprehensive geriatric assessment and evaluates mobility, physical activity, energy level, comorbidity burden, and functional dependence [33]. In geriatric oncology settings, the CFS has a prognostic value for predicting negative outcomes [34].

### 5.3. Comprehensive Geriatric Assessment (CGA): Components and Implementation

**Figure 1:**
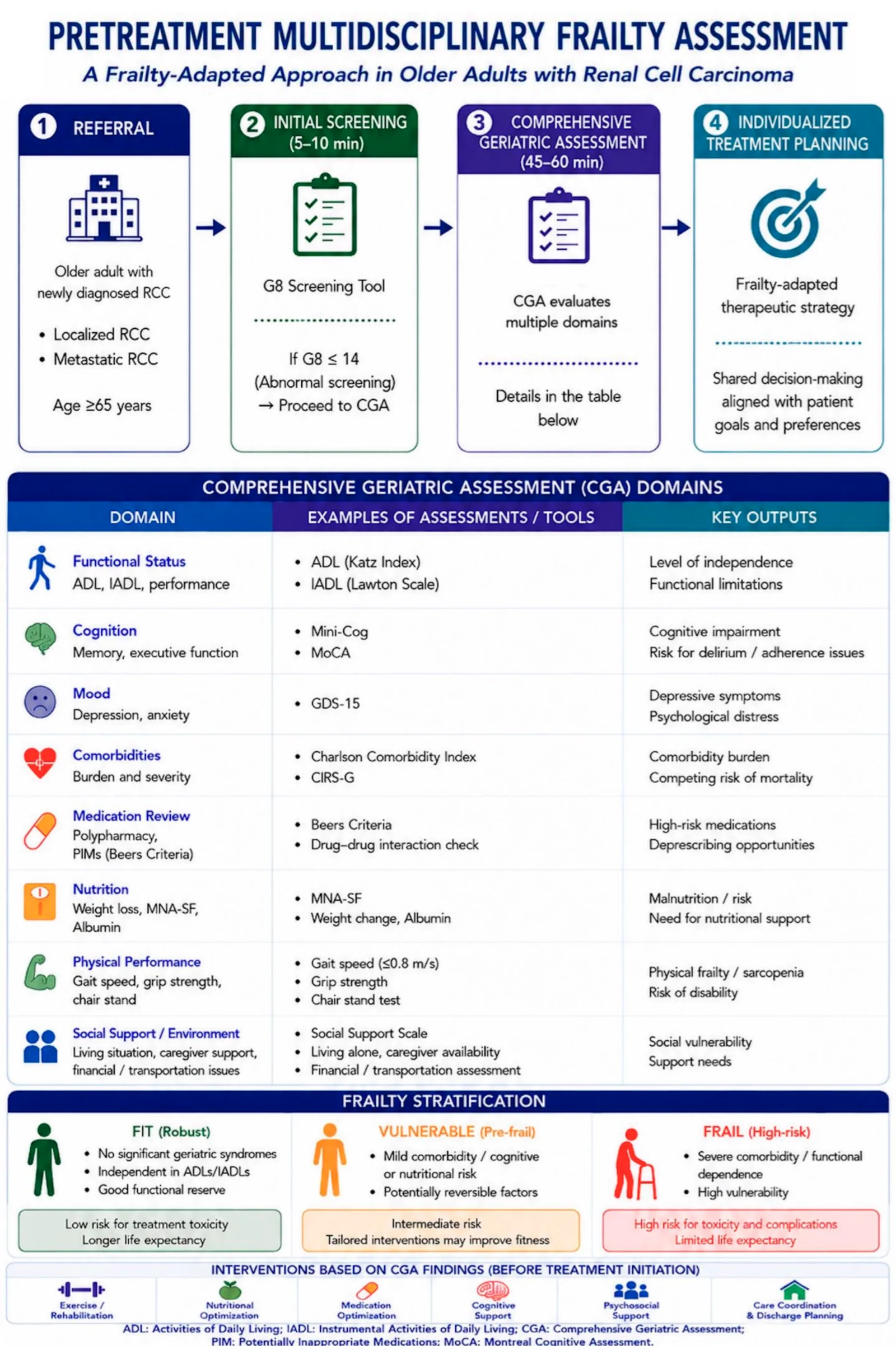
Pretreatment Multidisciplinary Frailty Assessment.

CGA is an interdisciplinary diagnostic process designed to assess medical, psychological, and functional capabilities in geriatric populations [35]. Within frailty-adapted RCC therapeutic paradigms, the CGA establishes the baseline for personalized treatment stratification, differentiating fit, vulnerable, and frail phenotypes via specific prognostic domains. The core CGA domains and representative assessment tools are summarized in Figure 1.

Implementation of the full CGA can be resource-intensive in a busy clinic. Consequently, to optimize clinical workflow in high-volume settings, a two-step algorithmic approach could be used: initial screening with abbreviated tools (such as the G8 or the Clinical Frailty Scale) followed by a full CGA for those identified as “at risk” [29,35].

### 5.4. Definition and Clinical Classification of Frailty (Fit/Vulnerable/Frail)

Frailty is a multidimensional geriatric syndrome characterized by decreased physiological reserve and impaired resistance to internal and external stressors, resulting from cumulative decline across multiple organ systems [36]. In older patients with RCC, frailty should not be equated with chronological aging alone. Rather, it reflects a state of biological vulnerability that may reduce tolerance of both the metabolic burden of malignancy and the toxicities of systemic anticancer therapy [32].

A recent meta-analysis demonstrated that frailty was independently associated with significantly worse overall survival and progression-free survival in patients with RCC [17]. Beyond traditional oncologic parameters, frailty appears to influence treatment tolerance, hospitalization risk, functional decline, and treatment discontinuation. Early evidence in metastatic RCC treated with immune checkpoint inhibitors suggests that the G8 screening tool can identify frail patients at higher risk of inferior survival outcomes; however, its prognostic performance may be weaker than disease-specific models that demonstrate more consistent discrimination for both overall and progression-free survival in selected cohorts [37].

In a frailty-adapted therapeutic paradigm, older patients with RCC may be stratified into fit, vulnerable, and frail phenotypes according to physiological reserve and validated screening tools followed by comprehensive geriatric assessment. Current data suggest that geriatric screening and assessment tools may be particularly useful for predicting severe treatment-related toxicities as well as important survival outcomes, including overall survival and progression-free survival [17,32].

For vulnerable patients, prehabilitation strategies—including exercise, nutritional optimization, and rehabilitation—may help improve functional reserve before treatment [38]. In frail patients, intensive treatment strategies may increase the risk of grade ≥ 3 treatment-related toxicity, hospitalization, and treatment intolerance, often leading to early dose reduction or discontinuation of systemic therapy [17,39]. However, the available evidence originates predominantly from heterogeneous cohorts, and RCC-specific data remain limited. Frailty-based treatment strategies are summarized in Table 2.

### 5.5. The Cancer and Aging Research Group (CARG) Score

Unlike general frailty tools, the CARG score was specifically developed to predict severe chemotherapy-related toxicity in older adults. The CARG model integrates both geriatric parameters and clinical variables including laboratory findings and treatment intensity. The final score is generated from 11 variables and stratifies patients according to their risk of grade 3–5 toxicities [40]. Recent studies suggest that the CARG score may outperform ECOG PS in predicting adverse events in patients receiving chemotherapy [41]. Although the CARG score has been validated for predicting chemotherapy-related toxicity, its applicability remains limited for the novel RCC treatments, including immune checkpoint inhibitors (ICIs) and tyrosine kinase inhibitors (TKIs). The pretreatment multidisciplinary frailty assessment is summarized in Figure 1.

## 6. Management of Localized RCC in Older Adults

In the geriatric population, therapeutic decision-making should balance oncologic benefit with preservation of renal function, functional independence, perioperative safety, and quality of life. Particularly in frail older individuals, the risk of mortality related to comorbid conditions may exceed the risk of cancer-specific mortality, emphasizing the importance of individualized treatment planning [17].

### 6.1. Surgical Management (Partial vs. Radical Nephrectomy)

The choice between partial nephrectomy (PN) and radical nephrectomy (RN) should not rely solely on chronological age, but rather on a multidimensional evaluation including physiological reserve, frailty status, baseline renal function, tumor complexity, comorbidity burden, and estimated life expectancy.

For decades, partial nephrectomy (PN) has been considered the standard treatment for T1 renal tumors because of its ability to preserve renal function. The randomized EORTC 30904 trial demonstrated similar long-term cancer-specific outcomes between PN and RN, although an overall survival advantage for nephron-sparing surgery was not observed [42].

In the prospective multicenter RECORD2 study, patients with limited life expectancy experienced significantly higher postoperative complication rates than those with longer life expectancy (20.6% vs. 9.9%; *p* < 0.001), demonstrating that physiological reserve and competing mortality, rather than chronological age alone, are major determinants of perioperative risk [43]. Likewise, the multicenter RESURGE collaborative study demonstrated that carefully selected patients aged ≥75 years could safely undergo PN with acceptable perioperative morbidity and preservation of renal function, although complication rates increased with frailty and surgical complexity [44]. The multicenter RECORD2 study externally validated the Martini nomogram for predicting significant postoperative renal function decline following partial nephrectomy, demonstrating good predictive performance, particularly for robotic and laparoscopic approaches. Such individualized risk prediction may be especially valuable in older patients, in whom preservation of renal function is a key treatment objective [45].

However, in the geriatric population, achieving the surgical “trifecta” of negative surgical margins, preservation of postoperative renal function, and avoidance of perioperative complications may sometimes be more challenging. Reduced baseline renal reserve, sarcopenia, frailty, polypharmacy, and increased perioperative vulnerability frequently complicate surgical recovery in older adults [44,46]. Compared with PN, RN is generally associated with shorter operative times and lower rates of procedure-specific urological complications, such as urinary leakage or postoperative hemorrhage. In frail older individuals with a healthy contralateral kidney and limited life expectancy, RN may therefore represent a pragmatic strategy to reduce perioperative morbidity [46,47].

Current evidence suggests that nephron-sparing surgery should remain the preferred option whenever technically and physiologically feasible in older patients. Nevertheless, the survival benefit associated with renal preservation may become less pronounced in selected individuals aged >75 years, especially when mortality from non-cancer comorbidities outweighs the long-term consequences of renal functional decline [4,48,49]. Key differences between PN and RN are summarized in Table 3.

### 6.2. Systemic Treatments After Surgical Management of Localized RCC

Adjuvant tyrosine kinase inhibitor studies, namely ASSURE, S-TRAC, PROTECT, ATLAS, and SORCE, did not demonstrate an OS benefit [50,51,52,53,54]. In contrast to adjuvant TKI therapy, ICIs have recently demonstrated efficacy in the adjuvant setting. In the KEYNOTE-564 study, adjuvant pembrolizumab significantly improved disease-free survival across clinically relevant subgroups, including older adults [55]. Nevertheless, in geriatric populations, the potential oncologic benefit of adjuvant immunotherapy should be carefully balanced against the risk of chronic or persistent immune-related toxicities, particularly endocrine dysfunctions that may permanently affect quality of life and long-term functional status [56,57]. Long-term KEYNOTE-564 results further supported adjuvant pembrolizumab in selected patients [58].

### 6.3. Active Surveillance (AS)

Active surveillance has emerged as an important management strategy for older patients with small renal masses (SRMs, ≤4 cm) [59]. Prospective observational registries, including the Delayed Intervention and Surveillance for Small Renal Masses (DISSRM) study, have shown that many SRMs demonstrate slow linear growth, often with median rates below 0.3 cm/year, although variability exists across cohorts [60,61]. Delayed treatment remains feasible in cases of accelerated growth, increasing tumor size, or radiologic progression, although many older patients may never require definitive intervention during their lifetime [5,61].

Importantly, active surveillance should be distinguished from watchful waiting. Active surveillance consists of structured radiological monitoring with predefined triggers for intervention, whereas watchful waiting reserves treatment for symptomatic progression and is generally appropriate only for patients with markedly limited life expectancy. In addition, renal mass biopsy should be considered in selected patients undergoing active surveillance when histological confirmation is expected to influence management decisions.

### 6.4. Stereotactic Body Radiotherapy (SBRT)

Stereotactic body radiotherapy (SBRT) has emerged as a promising non-invasive local treatment option for patients with localized RCC who are medically inoperable, have substantial comorbidities, or decline surgery. Recent prospective studies and systematic reviews have demonstrated local control rates exceeding 90%, with low rates of grade ≥ 3 toxicity and acceptable preservation of renal function following SBRT in carefully selected patients [62,63,64]. Although prospective data specific to older adults remain limited, SBRT represents an attractive alternative for frail older adults because it avoids anesthesia and minimizes peri-procedural morbidity. Current international recommendations consider SBRT an appropriate treatment option for selected patients who are unsuitable for surgery or thermal ablation; however, randomized comparative studies against established local therapies are still lacking [65].

### 6.5. Ablative Therapies

For older patients with localized RCC who are unsuitable for surgery because of frailty, comorbidity burden, or impaired physiological reserve, minimally invasive ablative therapies have emerged as important nephron-sparing treatment alternatives. The importance of obtaining histopathological confirmation by renal mass biopsy before local ablative therapies should be recognized despite the potential procedure-related complication risks. Of these approaches, cryoablation (CA) and radiofrequency ablation (RFA) are the most commonly utilized modalities in contemporary practice [66,67]. Cryoablation is generally associated with more favorable local oncologic control than radiofrequency ablation and has therefore become the preferred ablative modality in many tertiary centers [66].

Current evidence suggests that local recurrence and residual tumor rates may be higher after ablative therapies compared with PN. However, cancer-specific survival outcomes appear broadly comparable in appropriately selected patients with small localized tumors, particularly among frail older adults [68,69]. These procedures are frequently performed percutaneously under conscious sedation or limited anesthesia, thereby reducing perioperative morbidity and physiologic stress while maximizing preservation of renal parenchyma and postoperative renal function [70,71].

### 6.6. Treatment Selection Based on Life Expectancy and Frailty

Older patients classified as fit are generally appropriate candidates for standard surgical management, including PN for localized T1 tumors and radical nephrectomy. RN may be chosen for selected larger or anatomically complex masses when anticipated life expectancy supports the potential long-term oncologic benefit [4].

In vulnerable patients, less invasive strategies such as active surveillance or thermal ablation are frequently favored to minimize perioperative morbidity, reduce functional decline, and maintain overall physiological stability. In selected cases where surgery remains appropriate, prehabilitation interventions including nutritional optimization, physical exercise, and functional support may improve postoperative recovery and treatment tolerance [72].

In contrast, management of frail older adults with markedly reduced physiological reserve or limited life expectancy often prioritizes active surveillance, symptom control, and supportive care measures. In this population, competing non-cancer mortality frequently exceeds the oncologic risk associated with small localized renal masses, while aggressive intervention may add substantial treatment-related morbidity.

## 7. Systemic Therapy in Metastatic RCC

### 7.1. First-Line Treatment

#### 7.1.1. ICI-Based Combinations

The integration of ICIs with either a VEGF-TKI or another ICI has established a new standard of care for the first-line treatment of mRCC. In the geriatric population, these doublet regimens offer superior efficacy compared to TKI monotherapy. However, clinicians must carefully balance maximizing oncological response with managing the additive toxicity of two distinct drug classes [22].

*Pembrolizumab + Axitinib*

Pembrolizumab is widely used in combination with vascular endothelial growth factor receptor tyrosine kinase inhibitors (VEGFR-TKIs), including axitinib and lenvatinib.

The KEYNOTE-426 trial demonstrated sustained improvements in OS, PFS, and ORR compared with sunitinib, with durable benefit. Importantly, subgroup analyses suggested that the efficacy of the regimen was preserved across age groups, including patients aged ≥65 years [73].

Older adults appear particularly susceptible to VEGF-related toxicities such as hypertension, diarrhea, fatigue, and anorexia. Because axitinib has a relatively short half-life, temporary interruption or dose reduction in the TKI component is frequently effective in controlling adverse events without permanent discontinuation of pembrolizumab [74].

In older adults receiving combination therapy, it can be clinically challenging to differentiate VEGFR-TKI-induced toxicities (such as hypertension, fatigue, diarrhea, or hand–foot syndrome) from pembrolizumab-associated immune-related adverse events (irAEs), including colitis, hepatitis, pneumonitis, or endocrinopathies [75]. Because treatment-related symptoms may mimic baseline functional decline or symptoms related to multimorbidity or normal aging, identification of underlying irAEs may be delayed [56,57].

*Nivolumab + Cabozantinib*

Nivolumab plus cabozantinib demonstrated significant improvements in OS, PFS, and ORR compared with sunitinib [21].

Although subgroup analyses were not specifically powered for geriatric populations, patients aged ≥65 years appeared to derive efficacy benefits comparable to those observed in the overall study population [76].

Real-world evidence from the JK-FOOT consortium further suggests that nivolumab plus cabozantinib may represent a comparatively manageable option for older patients with mRCC. In this cohort, patients receiving nivolumab plus cabozantinib were older on average than those treated with pembrolizumab plus lenvatinib (median age 72 vs. 68 years), yet the regimen demonstrated a favorable safety profile with lower rates of severe treatment-related adverse events and treatment discontinuation [77].

From a geriatric oncology perspective, the use of cabozantinib at the 40 mg daily dose employed in the CheckMate 9ER regimen may be better tolerated than the 60 mg dose commonly used in monotherapy settings. Nevertheless, dose interruptions and reductions remain frequent in older or frail patients to mitigate fatigue, anorexia, hand–foot syndrome, hypertension, and functional decline. Individualized dose optimization is therefore particularly important in geriatric practice [21,77].

*Pembrolizumab + Lenvatinib*

Pembrolizumab plus lenvatinib demonstrated significant improvements in OS, PFS, and ORR compared with sunitinib [23]. The regimen achieved one of the highest response rates reported in frontline mRCC therapy [23]. However, toxicity data specifically in older populations are limited because subgroup analyses in older patients were exploratory rather than prospectively designed.

In the CLEAR trial, grade ≥ 3 treatment-related adverse events (TrAEs) occurred in more than 70% of patients receiving the combination treatment [23]. The most clinically relevant toxicities were hypertension, diarrhea, fatigue, and weight loss [23,78].

A 2026 JK-FOOT analysis reported higher rates of severe TrAEs and treatment discontinuation with pembrolizumab plus lenvatinib than with nivolumab plus cabozantinib [77]. Reduced starting doses or de-escalation strategies may be considered in selected vulnerable or frail older adults based primarily on expert opinion and real-world evidence [28,79,80].

*Nivolumab + Ipilimumab*

The combination of nivolumab and ipilimumab represents a standard first-line treatment option for patients with intermediate- or poor-risk mRCC, based on the results of the pivotal CheckMate 214 study [81].

Subgroup analyses from CheckMate 214 demonstrated that patients aged ≥65 years experienced overall survival and response benefits comparable to those observed in the overall trial population [81]. Emerging real-world evidence suggests that older patients had similar toxicity rates; however, frail patients may experience higher rates of treatment interruption or discontinuation secondary to immune-related toxicity and functional decline during dual checkpoint blockade therapy [82].

#### 7.1.2. VEGF-TKI Monotherapy

While these agents provide robust oncological control, their use in older adults is frequently limited by class-related toxicities that intersect with pre-existing age-related comorbidities, such as hypertension and renal insufficiency [83].

*Sunitinib*

VEGF-TKIs remain relevant treatment options in patients who are ineligible for immunotherapy-based regimens [84,85]. In older adults, these agents demonstrate efficacy outcomes comparable to those observed in younger populations; however, their distinct toxicity profiles are clinically important in treatment selection [86,87].

Sunitinib is more frequently associated with fatigue and hematologic toxicities, including neutropenia and thrombocytopenia, which may be particularly relevant in older patients with reduced physiological reserve [87]. Alternative dosing strategies, such as modified schedules (e.g., 2 weeks on/1 week off instead of the conventional 4/2 regimen), have been explored to improve tolerability while maintaining clinical efficacy [88].

*Pazopanib*

Pazopanib was noninferior to sunitinib with regard to PFS and OS. Moreover, health-related quality of life favored pazopanib over sunitinib [86]. In that regard, pazopanib was associated with lower rates of fatigue and hand–foot syndrome, which may be advantageous in selected older patients [86]. However, pazopanib is associated with a higher risk of hepatotoxicity, including transaminase elevation, necessitating regular liver function monitoring, particularly in patients with polypharmacy [85].

*Cabozantinib*

Cabozantinib improved PFS but not OS compared with sunitinib, with similar rates of treatment-related adverse events [89]. In clinical practice, cabozantinib is administered at a standard dose of 60 mg daily; however, dose reductions are frequently required due to treatment-related toxicity, particularly in older adults. Real-world data indicate that older patients are more likely to experience dose modifications and treatment interruptions due to adverse events such as fatigue, diarrhea, and anorexia. Although retrospective data suggest that dose reduction does not significantly compromise disease control, prospective evidence remains insufficient to support routine, upfront dose de-escalation strategies [90,91].

In geriatric populations, cabozantinib-related adverse effects, particularly weight loss and gastrointestinal toxicity, may contribute to accelerated sarcopenia and functional decline, thereby increasing vulnerability and potentially contributing to transitions from a fit to a frail state in susceptible patients [90,92,93].

### 7.2. Subsequent-Line Treatments

*Nivolumab*

Subgroup analyses from the CheckMate 025 study suggested that older patients, including those aged ≥75 years, derived overall survival benefits comparable to those observed in younger populations [94]. However, data regarding outcomes in patients aged ≥80 years remain limited, and the potential influence of immunosenescence on treatment efficacy continues to be investigated [56,95].

Available evidence indicates that the incidence of severe immune-related adverse events is not substantially increased in older adults receiving ICIs [95]. Nevertheless, even lower-grade toxicities such as fatigue, diarrhea, anorexia, or endocrinopathies may produce disproportionately greater functional decline, hospitalization risk, and deterioration in quality of life among frail older patients with limited physiologic reserve [96].

*Axitinib*

The AXIS trial demonstrated an improved PFS and ORR with axitinib compared to sorafenib. Hypertension, diarrhea, and fatigue were the most common grade ≥3 adverse events (AEs) in the axitinib arm [97].

Older patients treated with VEGFR inhibitors remain at increased risk of cardiovascular toxicity, predominantly manifesting as hypertension, which may be asymptomatic but clinically relevant. Accordingly, baseline cardiovascular risk assessment and regular monitoring during treatment are recommended [22,91].

*Cabozantinib*

In the METEOR trial, grade 3 or higher AEs occurred in 68% of patients receiving cabozantinib and were mostly managed with dose reduction [92,98]. A subsequent analysis of the METEOR trial evaluated efficacy and safety across the age-based subgroups (<65 years, 65–74 years, ≥75 years). According to this analysis, the PFS, OS and ORR benefits of cabozantinib were maintained across all age subgroups. Although the overall safety profile was similar between treatment arms across age groups, dose reductions and discontinuations were more common in patients aged ≥75 years [99].

*Tivozanib*

The TIVO-3 trial evaluated tivozanib in patients with heavily pretreated mRCC. The study demonstrated a significant improvement in PFS with tivozanib compared with sorafenib, along with a higher ORR and more durable responses. Importantly, tivozanib showed a more favorable tolerability profile, with fewer dose reductions, interruptions, and discontinuations compared with sorafenib [100]. A dedicated subgroup analysis evaluating age-related tolerability outcomes showed that the safety profile of tivozanib remained manageable in geriatric patients, with no unexpected toxicities [101].

*Everolimus*

Everolimus has clear clinical utility for selected older patients with mRCC, particularly those who are intolerant, ineligible, or refractory to first-line immunotherapy and anti-angiogenic regimens [102,103].

The RECORD-1 trial demonstrated that everolimus provided a PFS benefit across multiple age groups, including patients aged ≥65 and ≥70 years, with efficacy outcomes generally comparable to those observed in younger populations [102].

Noninfectious pneumonitis represents one of the most clinically significant complications and may be particularly challenging to recognize in frail older adults with underlying pulmonary disease or cardiopulmonary comorbidity, where symptoms may overlap with infection or heart failure [104,105].

Metabolic toxicities, including hyperglycemia and hyperlipidemia, occur frequently during everolimus therapy and may complicate the management of older patients with pre-existing diabetes mellitus, metabolic syndrome, or cardiovascular disease. Careful interdisciplinary monitoring is recommended during treatment [104,106].

Stomatitis is another common dose-limiting toxicity that may contribute to impaired oral intake and lead to dehydration, weight loss, and functional decline in vulnerable geriatric patients [104,107].

### 7.3. Emerging and Novel Agents: Belzutifan

The phase III LITESPARK-005 trial showed that belzutifan significantly improved PFS and ORR compared with everolimus in patients with previously treated advanced clear-cell renal cell carcinoma (ccRCC) who had received first-line ICI and VEGF-targeted therapies [108]. Updated analyses presented in 2024 and 2025 confirmed the durability of clinical benefit across multiple patient subgroups, including older adults, although geriatric-specific analyses remain limited [108,109].

Belzutifan may be a promising option for selected vulnerable older patients because of its comparatively lower incidence of classic TKI-associated toxicities such as severe hypertension, hand–foot syndrome, and gastrointestinal toxicity. On the other hand, the favorable toxicity profile of belzutifan should be balanced against limited data in the geriatric population. Furthermore, its distinct side effect profile, particularly anemia and hypoxia, requires careful monitoring in these patients [109,110].

Clinical trials have reported anemia in the majority of treated patients, with grade ≥ 3 anemia occurring frequently enough to necessitate dose interruptions, erythropoiesis-stimulating agents, or blood transfusions in a substantial subset of patients [108,111]. In frail older adults, treatment-related anemia may exacerbate fatigue, sarcopenia, reduced exercise tolerance, and underlying cardiovascular disease.

Hypoxia represents another clinically important toxicity. Although severe hypoxia is relatively uncommon, older adults with impaired pulmonary reserve or significant cardiopulmonary comorbidity may be particularly vulnerable to symptomatic oxygen desaturation. Accordingly, close clinical monitoring is recommended during therapy initiation and follow-up [110,111].

In addition to single-agent belzutifan, the combination of belzutifan with other TKIs such as lenvatinib has been evaluated in phase III trials. The LITESPARK-011 trial demonstrated that belzutifan plus lenvatinib was superior to cabozantinib in patients who had previously been treated with ICIs. Additionally, the rate of grade ≥ 3 AEs did not differ significantly, and no new safety signal was observed [112].

## 8. Practical Clinical Recommendations

Given the marked heterogeneity of the geriatric population, chronological age alone should not determine treatment intensity or eligibility. Instead, therapeutic decision-making should be guided by multidimensional geriatric assessment and individualized risk–benefit evaluation [28,113]. A geriatric multidisciplinary evaluation should begin with systematic frailty screening in all patients aged ≥65 years using validated tools such as the G8 and the Clinical Frailty Scale [32,33,35].

Integration of CGA findings into treatment planning is essential and ideally should occur within a multidisciplinary tumor board involving medical oncologists, urologists, radiation oncologists, geriatricians, dietitians, and supportive care specialists [113]. Fit older adults may tolerate standard ICI-based combinations similarly to younger patients, whereas vulnerable or frail individuals often require modified therapeutic strategies emphasizing tolerability and maintenance of functional status [114]. Current treatment strategies are summarized in Table 4 together with the corresponding landmark trials and levels of evidence.

Given the limited data, no validated RCC-specific frailty-tiered starting-dose algorithm currently exists for ICI–TKI combinations. Therefore, the treatment approach should primarily follow regimen-specific efficacy and toxicity data, organ function, drug–drug interactions, and geriatric assessment findings, while early toxicity surveillance and prompt label-directed dose interruption or reduction remain central to management.

Retrospective studies suggest that reduced starting doses of agents such as sunitinib or cabozantinib may improve treatment adherence, relative dose intensity, and duration of therapy without substantially compromising PFS or OS [88,90,93].

Older patients with RCC require closer and more comprehensive monitoring than younger populations, as treatment-related decline may initially manifest through subtle functional deterioration rather than laboratory abnormalities alone. Serial assessment of gait speed, grip strength, weight loss, nutritional status, and performance status may provide early indicators of treatment intolerance or evolving frailty [113]. Patient-reported symptom monitoring may further facilitate early detection of treatment-related adverse events [117].

## 9. Future Directions and Limitations

Future clinical research is expected to move beyond chronologic age and incorporate frailty-based stratification using validated tools to better evaluate treatment tolerability and outcomes in fit versus vulnerable patients. In parallel, there is a growing emphasis on patient-centered endpoints, including functional progression-free survival, maintenance of ADLs, cognitive preservation, and health-related quality of life. Future therapeutic strategies are also likely to explore adaptive treatment approaches, including modified or intermittent TKI dosing schedules and “stop-and-go” regimens to reduce treatment burden in older adults [118].

Emerging biomarkers of immunosenescence and biological aging may improve prediction of treatment efficacy and immune-related toxicity beyond chronologic age alone. Noninvasive monitoring strategies using circulating tumor DNA (ctDNA), urinary biomarkers such as Kidney Injury Molecule-1 (KIM-1), and inflammatory or nutritional indices including systemic immune–inflammation index (SII) may further refine risk stratification and therapeutic monitoring in older patients with limited physiologic reserve [119,120]. In summary, real-world studies should aim to bridge the gap between controlled clinical trials and routine geriatric oncology practice by helping to validate the safety and efficacy of novel treatment combinations in vulnerable or underrepresented populations often excluded from clinical trials.

This review has several limitations. First, because it was designed as a narrative review, no formal systematic-review methodology or risk-of-bias assessment was performed. Second, definitions of older age and frailty varied substantially across the included studies, limiting direct comparison between cohorts. Third, older and frail adults remain underrepresented in pivotal RCC trials, and much of the available evidence is derived from age-based subgroup analyses, retrospective studies, and real-world cohorts rather than prospectively designed geriatric-specific trials. In addition, commonly used geriatric tools have limited RCC-specific validation. Therefore, frailty-adapted treatment recommendations should be interpreted as a clinical decision-support framework.

## Figures and Tables

**Table 1:** Summary of Pharmacokinetic and Pharmacodynamic Alterations in Older Adults [18].

Physiological Parameter	Age-Related Change	Impact on RCC Therapy
Hepatic Function	↓ Liver blood flow and mass	Reduced CYP450 metabolism; potential for increased drug toxicity.
Renal Function	↓ GFR and tubular secretion	Delayed excretion of drugs; increased risk of nephrotoxicity.
Total Body Water	↓ Intracellular water	Higher peak concentrations of water-soluble agents.
Body Fat	↑ Adipose tissue	Increased volume of distribution for lipophilic drugs; prolonged half-life
Serum Albumin	↓ Albumin levels	Increased free (active) fraction of highly protein-bound drugs.
Gastrointestinal	↑ Gastric pH/↓ Motility	Potential for altered absorption of oral tyrosine kinase inhibitors (TKIs).

↑, increase; ↓, decrease.

**Table 2:** Frailty classification and therapeutic strategies.

Classification	Clinical Profile	Therapeutic Strategy
Fit	No significant geriatric syndromes; independent in all IADLs/ADLs; minimal comorbidities.	Standard of care (e.g., full-dose combination therapies) as per clinical guidelines.
Vulnerable	Mild deficits (e.g., mild cognitive impairment or nutritional risk); potentially reversible impairments.	Personalized treatment with proactive supportive care.
Frail	Severe deficits in domains; dependent for ADLs; high burden of multimorbidity; limited life expectancy.	Prioritize quality of life; consider individualized palliative care or best supportive care.

**Table 3:** Partial Nephrectomy vs. Radical Nephrectomy.

Feature	Partial Nephrectomy (PN)	Radical Nephrectomy (RN)
Oncologic control for T1 tumors	Excellent	Excellent
Renal function preservation	Superior	Inferior
Operative complexity	Higher	Lower
Operative time	Usually longer	Usually shorter
Risk of urine leak/reconstruction issues	Higher	Lower
Risk of chronic kidney disease progression	Lower	Higher
Frailty-adapted role	Best suited to fit patients and selected vulnerable patients after optimization.	Consider in fit or vulnerable patients when PN would add excessive operative risk; frailty alone is not an indication.
Tumor size/complexity	Preferred for cT1a (≤4 cm) and selected cT1b (>4–7 cm) tumors with low-to-moderate RENAL and/or PADUA complexity.	Preferred for cT2 (>7 cm) or high-complexity tumors (high RENAL and/or PADUA score) or when PN is not technically feasible.

**Table 4:** Summary of RCC Treatment Strategies and Landmark Trials.

Disease Setting	Treatment Strategy	Landmark Trial	Key Reference	Principal Findings	Evidence Level (OCEBM) *
Localized RCC	Partial nephrectomy	EORTC 30904	[42]	Preferred nephron-sparing approach for appropriately selected fit older adults with preservation of renal function.	2
	Active surveillance	DISSRM Registry	[60]	Appropriate for carefully selected frail patients with small renal masses and limited life expectancy.	3
	Thermal ablation	Review of focal ablation studies	[66]	Effective nephron-sparing alternative for medically inoperable patients.	3
Adjuvant therapy	Pembrolizumab	KEYNOTE-564	[55]	Improved disease-free survival and later demonstrated overall survival benefit after nephrectomy.	2
First-line metastatic RCC	Pembrolizumab + Axitinib	KEYNOTE-426	[22,115]	Improved OS, PFS, and ORR versus sunitinib.	2
	Pembrolizumab + Lenvatinib	CLEAR	[23]	Highest ORR and significantly prolonged PFS versus sunitinib.	2
	Nivolumab + Cabozantinib	CheckMate 9ER	[21]	Improved OS, PFS, ORR, and quality of life.	2
	Nivolumab + Ipilimumab	CheckMate 214	[81]	Durable overall survival benefit and higher complete response rates.	2
Subsequent therapy	Cabozantinib	METEOR	[92,98]	Improved PFS and OS compared with everolimus.	2
	Tivozanib	TIVO-3	[100]	Improved progression-free survival compared with sorafenib.	2
	Belzutifan	LITESPARK-005	[108]	Improved PFS and ORR versus everolimus following prior PD-L1 inhibitor and VEGF-targeted therapy.	2
	Belzutifan + Lenvatinib	LITESPARK-011	[112]	Significantly improved PFS and ORR versus cabozantinib after prior PD-(L)1 therapy; OS data remain immature.	2

* Levels of Evidence: Evidence levels were assigned according to the Oxford Centre for Evidence-Based Medicine (OCEBM) 2011 Levels of Evidence [116].

## Data Availability

No new data were created or analyzed in this study. Data sharing is not applicable to this article.

## Abbreviations

The following abbreviations are used in this manuscript:

ADL	Activities of Daily Living
AE	Adverse event
AS	Active surveillance
CARG	Cancer and Aging Research Group
ccRCC	Clear-cell renal cell carcinoma
CFS	Clinical Frailty Scale
CGA	Comprehensive Geriatric Assessment
CIRS-G	Cumulative Illness Rating Scale—Geriatrics
ctDNA	Circulating tumor DNA
DISSRM	Delayed Intervention and Surveillance for Small Renal Masses
ECOG	Eastern Cooperative Oncology Group
EORTC	European Organisation for Research and Treatment of Cancer
G8	Geriatric 8 screening tool
GDS-15	15-item Geriatric Depression Scale
HIF-2α	Hypoxia-inducible factor 2 alpha
IADL	Instrumental Activities of Daily Living
ICI	Immune checkpoint inhibitor
irAE	Immune-related adverse event
KIM-1	Kidney injury molecule-1
KPS	Karnofsky Performance Status
MeSHs	Medical Subject Headings
MNA	Mini Nutritional Assessment
MNA-SF	Mini Nutritional Assessment—Short Form
MoCA	Montreal Cognitive Assessment
mTOR	Mammalian target of rapamycin
OCEBM	Oxford Centre for Evidence-Based Medicine
ORR	Objective response rate
OS	Overall survival
PD-1	Programmed death-1
PD-L1	Programmed death-ligand 1
PFS	Progression-free survival
PROM	Patient-reported outcome measure
QoL	Quality of life
SBRT	Stereotactic body radiotherapy
SII	Systemic immune–inflammation index
TKI	Tyrosine kinase inhibitor
TrAE	Treatment-related adverse event
VEGF	Vascular endothelial growth factor
VEGFR	Vascular endothelial growth factor receptor
